# Nano- and Microplastics Migration from Plastic Food Packaging into Dairy Products: Impact on Nutrient Digestion, Absorption, and Metabolism

**DOI:** 10.3390/foods12163043

**Published:** 2023-08-14

**Authors:** Tafadzwa Kaseke, Tamara Lujic, Tanja Cirkovic Velickovic

**Affiliations:** 1 Center of Excellence for Molecular Food Sciences, Department of Biochemistry, University of Belgrade, Studentski trg 16, 11000 Belgrade, Serbia; 2 Department of Food Technology, Safety, and Health, Faculty of Bioscience Engineering, Ghent University, 9000 Ghent, Belgium; 3 Center for Food Chemistry and Technology, Ghent University Global Campus, Incheon 21985, Republic of Korea; 4 Serbian Academy of Sciences and Arts, Knez Mihajlova 35, 11000 Belgrade, Serbia

**Keywords:** nano- and microplastics, plastic food packaging, dairy products, nutrient digestion, absorption, metabolism, plastic oligomers

## Abstract

The ongoing use of plastic polymers to manufacture food packaging has raised concerns about the presence of nano- and microplastics (NMPs) in a variety of foods. This review provides the most recent data on NMPs’ migration from plastic packaging into dairy products. Also discussed are the possible effects of NMPs on nutrient digestion, absorption, and metabolism. Different kinds of dairy products, including skimmed milk, whole liquid milk, powder milk, and infant formula milk, have been found to contain NMPs of various sizes, shapes, and concentrations. NMPs may interact with proteins, carbohydrates, and fats and have a detrimental impact on how well these nutrients are digested and absorbed by the body. The presence of NMPs in the gastrointestinal tract may impact how lipids, proteins, glucose, iron, and energy are metabolized, increasing the risk of developing various health conditions. In addition to NMPs, plastic oligomers released from food packaging material have been found to migrate to various foods and food simulants, though information regarding their effect on human health is limited. Viewpoints on potential directions for future studies on NMPs and their impact on nutrient digestion, absorption, and health are also presented in this review.

## 1. Introduction

Human exposure to nano- and microplastics (NMPs) is a reality, and the contamination of food with NMPs has become an issue worldwide. As such, NMPs have attracted the attention of the entire world, including governments, non-governmental organizations, the scientific community, the public, and the media [1]. In addition, NMPs have become a topical issue in food production, processing, and packaging. Plastic particles smaller than 5 mm in diameter are referred to as microplastics (MPs), and those that are less than 1 μm in diameter are nanoparticles (NPs), according to the ISO definition [2]. Terminology has not been agreed upon yet; therefore, NPs are often referred to as particles of sizes less than 100 nm, and particles of sizes from 100 nm up to 1 µm are referred to as small microplastics [2]. NPs are generated from MPs due to the impact of environmental factors such as sunlight, water, temperature, and physical stress. Due to their larger specific surface area and volume ratio compared to those of MPs, NPs are more reactive and easily heteroaggregrate with organic matter and natural solids, interact with light, and are more harmful to biological organisms than MPs [3]. The analytical challenges of MNPs determination in biological tissues, particularly NPs, hampers our understanding of the full range of our exposure to MNPs. Most of the human exposure data and occurrence in foods are generated on MPs due to the lack of suitable analytical tools for NPs determination. However, progress made regarding the production of modeled NPs of different chemistry and properties, including dopped particles, has paved the way for studying their biodistribution and various in vitro effects [4], further prompting research efforts aiming at the detection and characterization of NPs in complex matrices, such as foods and biological tissues.

Plastics are preferred to other packaging materials due to their low production costs, easy transportability, versatility, lightweight nature, durability, and recycling potential [5]. Statistics from the Plastic Europe report have shown that plastic-based food packaging materials occupy the largest share of packaging materials [6], despite the global call to reduce plastic usage in food packaging and preparation. Studies continue to report NMPs in both processed and unprocessed foods, and therefore, NMPs have become a matter of concern all over the world. NMPs have been observed in drinking water (bottled and tap), beverages (alcoholic and non-alcoholic), honey, salt, sugar, ready-to-eat foods, sea foods, meat, and dairy products. 

Dairy products are some of the most consumed food products, given their richness in nutrients such as protein, minerals (calcium, magnesium, potassium, zinc, and phosphorus), and vitamins such as vitamin D, which play a key role in healthy human nutrition and development [7]. However, NMPs of diverse sizes, morphologies, and concentrations have been observed in dairy products [8]. Furthermore, NMPs were reported in plastic bottles used for infant milk preparation [9]. Actually, infants are more exposed to NMPs than any other age group, considering their daily milk consumption patterns [9]. The contamination of milk products with NMPs may occur at various stages along the value chain, from the farm to processing and packaging [10]. 

Due to the overwhelming evidence from the literature on the existence of NMPs in foods, the consumption of these plastic particles is inevitable, and therefore, food intake is one of the main pathways for NMPs to enter the human body. This has prompted researchers to further study the potential effect of NMPs on nutrient digestion. In vitro gastrointestinal digestion has revealed that NMPs negatively affect the digestion of lipids and starch [11,12]. Nonetheless, the effect of NMPs on nutrient digestion still remains understudied, particularly for nutrients such as proteins. Following oral exposure to NMPs, hazardous chemicals can be released in the gastrointestinal tract (GIT), leading to toxicity. Of particular concern are plastic oligomers (particularly cyclic oligomers of polyethylene terephthalate), side products of plastic production, recently identified as non-intentionally added substances of foods (NIAS). Given its role in nutrients digestion and absorption, the GIT has become the main target organ for NMPs, and studies have revealed that NMPs may cause nutrient absorption disorders depending on exposure and susceptibility [13]. Despite this, evaluating the NMPs’ risks to human health remains a challenge due to the particles’ complex compositions, variable sizes, and shapes [14].

In view of the above, this review aimed to discuss information reported between 2011 and 2023 on NMPs’ and their oligomers’ migration from plastic packaging into dairy products. In addition, the potential effects of NMPs on nutrient digestion, absorption, and related health problems are discussed.

## 2. Effect of NMPs on Human Health—Overview

NMPs may enter the human body through various routes, ingestion being the most relevant route for food and beverages contaminated with NMPs. The presence of NMPs in the GIT allows them to reach different parts of the body, where they may have both physical and chemical effects [13]. Despite the lack of definitive evidence linking NMPs to human health, various in vivo and in vitro studies have allowed conclusions to be drawn on the potential impact of NMPs on human health. NMPs have been associated with causing inflammation, disrupting the endocrine system, and disturbing gastrointestinal functions [12,15,16]. If NMPs pass through the intestinal barrier into the circulatory system, they may cause damage to important organs such as the liver and spleen. Respiratory tract irritation, obesity, asthma, cardiovascular diseases, and GIT diseases may be some of the possible health implications of NMPs [17]. Chemical additives to NMPs are also implicated in the health effects of NMPs [15]. More rigorous studies are warranted to explore the potential risks and health effects of this novel food and environmental contaminant. It should be highlighted that a thorough understanding of the influence of NMPs on human health depends on various factors, including the type of polymer, particle size, particle shape, concentration, exposure time, and types of chemical additives [15]. In addition, knowledge on mechanisms of absorption, distribution, and metabolism provides a clearer elaboration of the impact of NMPs on human health.

## 3. Bibliometric Analysis

**Figure 1:**
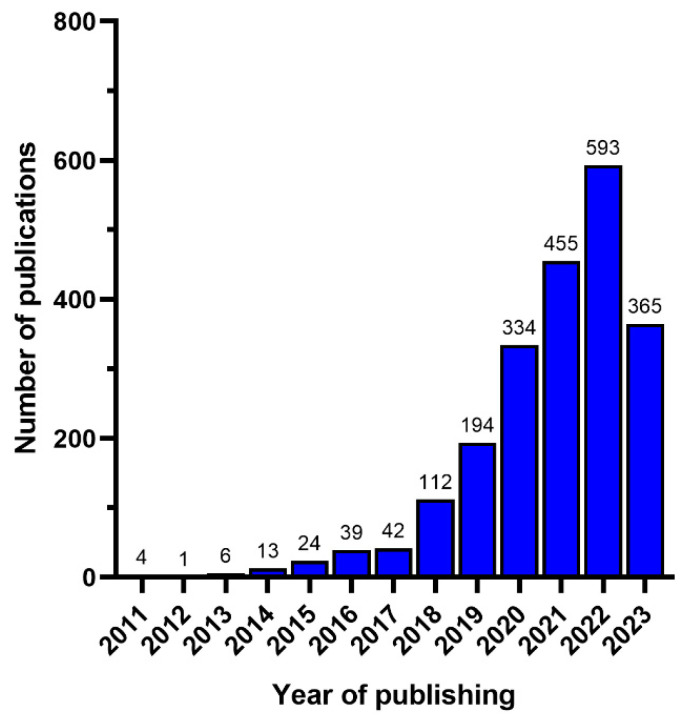
Annual publications from 2011 to 2023 searched in Web of Science on NMPs and oligomers in food and food simulants.

Articles, reports, and book chapters used to write this review were searched on the Google Scholar database and Web of Science. Information was also accessed over the internet. To broaden the search, the Boolean operators ‘AND’ and ‘OR’ were used. The keywords used for searching were nanoplastics, microplastics, dairy products, food packaging, nutrient digestion, human health, and plastic oligomers. The search mainly focused on scientific indexed papers and reports. All the articles were written in English. An increasing trend in the number of articles on NMPs and oligomers occurrence in food and drinking water has been observed in the past decade. Therefore, the scientific articles, reports, and book chapters used in this review were published between 2011 and 2024, with more than 90 percent of the publications being less than four years old (Figure 1). About 2187 articles were found in the literature, and after screening, only 104 articles were finally used to write this review. The key words ‘nano- and microplastics’; ‘human health’; ‘plastic oligomers‘; ‘dairy products’; and nutrient digestion’ appear either in the title or abstract. Websites of organizations with an interest in NMPs in food, including the Food and Agriculture Organization (FAO) and European Food Safety Authority (EFSA), were also explored. The findings of this review were used to develop suggestions for further research and mitigation.

## 4. Types of Polymers Used as Packaging in the Food Industry and Chemical Additives

Food packaging plays a crucial role in the food industry, as it facilitates the handling, transport, storage, and quality preservation of food. Nonetheless, food is always in direct contact with packaging; therefore, the safety of plastic packages regarding food should be prioritized. The most common polymers used in food packaging include polyethylene (PE), polyethylene terephthalate (PET), high-density polyethylene (HDPE), low-density polyethylene (LDPE), polypropylene (PP), polyvinyl chloride (PVC), and polystyrene (PS) (Table 1). In addition, take-out containers, trays, and plastic film contain these polymers, which are the major sources of NMPs’ release into food. NMPs may be introduced into food from packaging material through harsh environmental conditions, release from newly manufactured packaging, packaging contamination during the manufacturing process, mechanical force, a loose structure, and a rough surface [13]. The possibility of NMPs being introduced through the raw materials should not be completely ruled out. In addition to NMPs contamination, the migration of the chemical components of the polymers into food is a huge concern. 

Polymers used in food packaging are advertised as ‘green’ and non-toxic and used to manufacture different types of packaging materials (Table 1). However, under extreme conditions (high temperatures, UV light, and changes in pH), polymers may undergo certain physico-chemical alterations; thus, residual monomers, oligomers, and chemicals such as plasticizers, flame retardants, pigments, antimicrobial agents, heat stabilizers, UV stabilizers, fillers, and flame retardants, added to the plastic packaging (Table 1), may be released into the food, causing food safety issues [18]. Chemical additives are added to enhance the functional properties of the plastic packaging. The migration of plastic packaging chemical additives is partly due to the lack of covalent bonds between them and the polymers. Also, factors such as the food type, contact time, and quality of the plastic material are implicated. According to Smith et al. [19], the continued degradation of plastic increases the surface-area-to-volume ratio, promoting the leaching of chemical additives. 

The migration of styrene, the main component of PS, was observed in different food simulants (10, 50, 95% ethanol and 3% acetic acid) (0.110–6 µg/mL [20]), meat products (0.4–160 ng/g [21]), and dairy products (yoghurt and cream, 5–30 µg/kg [22]). Styrene is considered a toxic compound and is currently under monitoring by the European Food Safety Authority. Its application could soon be restricted, especially in food packaging destined for European markets [22]. He et al. [18] studied the migration of chemical additives from microwavable plastic containers using different food simulants including 10% distilled water, 3% ethanol, 3% acetic acid, and 50 and 95% ethanol and detected chemicals including isomers of hexadecanamide and oleamide, PEG oligomers of N,N-bis(2-hydroxyethyl) alkyl(C8–C18)amines, and Irgafos 168 OXO at concentrations ranging from 0.02 to 14.90 µg/kg. Other chemicals including titanium dioxide, ATBC (O-acetyl tributyl citrate), phthalate (DEP and DEHP), DEHA, ATBC, diisooctyl phthalate (DIOP), polyethylene glycol, phthalic anhydride, and stearamide were detected in corn snacks, cookies, cake, bottled water, alcoholic beverages, meat, and dairy products [15]. Evidence on the harmful effects of chemical additives in food plastics is still limited due to challenges such as the lack of standards for identifying the chemical additives [15,23]. 

**Table 1:** Common types of polymers, chemical additives, applications in the food industry, and potential health effects.

Type of Polymer	Application	Type of Monomer	Common Chemical Additives	Potential Health Effects of Chemical Additives	References
Polypropylene (PP)	Food packaging, sweet and snack wrappers, hinged caps	Propylene	Irgafos 168 (tris (2,4-di-tert-butylphenyl) phosphite) and Polystyrene-b-poly (ethylene-co-butylene)-b-polystyrene (SEBS)	Irgafos 168 is associated with cell growth inhibition and endocrine disruption	[24,25]
High-density polyethylene (HDPE)	Milk bottles	Ethylene	Calcium stearate, halogens, and metal catalysts.	No reported adverse health effects	[24,26]
Low-density polyethylene (LDPE)	Food packaging film, food containers, and trays	Ethylene	Calcium stearate, halogens, and Irgafos 168.	Irgafos 168 is associated with cell growth inhibition and endocrine disruption.	[24,27,28]
Polystyrene (PS)	Dairy and fishery food packaging, bottle caps, cups, and trays	Styrene	Hexabromocyclododecane	Hepatotoxicant, endocrine disruptor, and neurotoxic.	[26,29]
Polyethylene terephthalate (PET)	Water, soft drink, and juice bottles	Terephthalic acid and ethylene glycol	Benzothiazole, triphenyl phosphate (TPhP), Irgafos 168 (tris (2,4-di-tert-butylphenyl) phosphite), tripropyl phosphate (TPP), phthalide, tris (2-chloroethyl) phosphate (TCEP), benzophenone, and phthalimide	Neurotoxic (neuroinflammation and neuronal apoptosis); induces lipid peroxidation; causes lysosomal dysfunction, thyroid endocrine disruption; carcinogenic; hepatotoxic; and causes developmental disfunction.	[26,28,30]
Polyvinyl chloride (PVC)	Trays, bottles, containers, flexible films, caps, and can linings	Vinyl chloride	Plasticizers: dioctylphthalate esters; UV-stabilizers: barium, lead oxide, or cadmium carboxylates; Pigments: carbon black, titanium dioxide, organotin stabilizers, barbituric acid or thiobarbituric acid, dialkyltin maleates, 2-Benzimidazolylacetonitrile, naphthalene, and diisonyl phthalate.	Plasticizers may cause reproductive and developmental disorders, endocrine disruption, and cardiometabolic diseases. UV-stabilizers may cause oxidative stress and acute hepatotoxicity. Pigments may cause inflammation.	[25,26,31,32]

## 5. Analytical Methods for the Detection of NMPs and Plastic Oligomers 

According to most publications, there are several steps that must be considered and carefully monitored when analyzing the content of NMPs. Usually, these include sample collection, sample preparation/particle extraction, and quantification. Since plastic particles are ubiquitous, the preparation of appropriate controls is crucial for mitigating contamination and the overestimation of particle content.

During the sample preparation, filtration, digestion, and density separation are commonly used methods. Regarding dairy samples, a number of these methods have been employed. Kutralam-Muniasamy et al. [8] used only a warm filtration step and analyzed the sample MPs directly from the filter. However, dairy samples can be prepared using a digestion step, which includes the use of strong bases [33], a combination of enzymes and strong bases [10,34], and oxidative agents such as hydrogen peroxide [35] (Table 2).

As for sample analysis, multiple techniques can be used. The most common methods are based on spectroscopy or GC/MS in combination with pyrolysis or thermal extraction and desorption (TAD). Methods based on spectroscopy provide the chemistry, size, and shape of the MPs (Raman, µFTIR, FPA–µFTIR) but cannot determine plastics of sizes less than 10 µm (i.e., suitable only for MPs) nor estimate the mass of the plastic. GC/MS-based methods generate aggregate data on MNPs, so there is uncertainty regarding the contribution of NPs in the total MNP mass reported by these methods. A novel way of separating and characterizing particles <1 µm uses flow-based techniques such as asymmetrical flow field flow fractionation (AF4) combined with different detectors. There are plenty of excellent recent reviews on methods used in NMP characterization [36,37,38,39]. 

The approach to detecting and quantifying plastic oligomers is very complex. Samples can be analyzed directly without preparation, after dissolving the polymer, or after the dissolution and subsequent precipitation of the polymer. Alternatively, an extract of the polymer or migrants to a food simulant can be analyzed. The most used technique for oligomer identification and quantification is mass spectrometry coupled with liquid chromatography. However, this is especially challenging because of the large number of molecules that can be formed and the limited number of commercial standards available, particularly of linear oligomers, making the quantification of most molecules semiquantitative [23].

## 6. Migration and Occurrence of NMPs in Dairy Products

Among other types of foods, the contamination of milk products with NMPs has been reported. According to the literature, NMPs have been observed in diverse types of dairy products, including skimmed or whole liquid and powder milk, infant formula milk, and other dairy products such as yoghurt (Table 2). Depending on the type of analytical methods used, the occurrence data are reported as particle numbers or the mass of plastics. 

MPs amounting to 16.2 million per liter were detected in prepared formula milk [9]. The authors estimated that infants (<12 months old) may consume approximately 1.6 million MPs per day. The temperature of the water used to prepare the formula milk and the sterilization process were implicated in the migration of MPs [9]. Common plastics used to package breastmilk (PE, PET, and nylon 6) were reported to release MPs of varied shapes (irregular and oval), weights (0.22 and 0.47 mg), and sizes (<0.8 μm) [40]. The authors estimated that infants may ingest about 0.61–0.89 mg/day from the plastic materials used to pack milk. Kutralam-Muniasamy et al. [8] investigated MPs’ occurrence in whole and lactose-free milk and observed MPs of <11 µm in size varying from 3 to 11 particles per liter (Table 2). A study on boxed milk powder and canned milk powder showed that the plastic packaging in the boxed milk powder was implicated in the release of MPs into the milk powder [41]. In this study, the boxed milk powder contained eightfold higher MPs than the canned milk powder (Table 1). Another interesting finding from the study of Zhang et al. [41] was that MPs from feed bottles were more than sixfold higher than those from milk powders. These results suggest that the stress applied to the bottles during milk preparation and cleaning had a significantly positive effect on the generation of MPs. 

Very small sizes (<5 µm) and amounts ranging from 204 to 1004 MPs/100 mL were observed in raw milk collected soon after milking and powdered cows’ milk products [10]. Among the different polymers, PE was the most frequently found polymer, accounting for 31% of the MPs, compared to PP and polyester (PES), which contributed 27 and 23%, respectively. PVC, PP, and PE-based MPs (2–12 µm) were also detected in breastmilk [33]. The authors observed no significant correlation between the MPs data and the patients’ food consumption patterns and suggested that the patients ingested the MPs through various sources. Their findings confirmed the ubiquitous nature of NMPs. Skimmed milk samples collected from different cities in Ecuador and packed in PE containers contained MPs (2.48–183.37 µm; both fragments and fibers), ranging from 16 to 53 MPs per liter [35]. The presence of MPs in the milk samples was suggested as the result of milk processing methods. Other studies, which reported the occurrence of MPs in dairy products, are shown in Table 1. Given the wide use of milk to produce infant food products, the occurrence of MPs in dairy products raises serious concerns.

**Table 2:** Migration of MPs into dairy products.

Type of Milk Product	Type of Packaging Material	Country of Study	Sample Processing and MPs Characterization			MPs’ Shape and Size	Quantity of MPs	References
			MPs’ Extraction	Filter Pore Size	Polymer Characterization			
Skimmed milk	Polyethylene	Ecuador	Filtration and digestion with 30% H_2_O_2_ for 72 h	250 µm	FTIR	Fibers and fragments (2.48–183.37 µm)	16–53 MPs/L	[35]
Whole, half fat, light, and lactose-free milk	Polysulfone	Mexico	Filtration (11 μm filter pore size)	11 µm	Nikon epifluorescence microscope H6000L; SEM-EDS; Raman spectroscope	Fibers and fragments (>11 µm)	3–11 MPs/L	[8]
Milk powder	Boxed with inner plastics	China	Enzyme digestion (gastric juice containing pepsin followed by the addition of a pancreatin enzyme) and filtration	8 µm	FTIR	Fibers and fragments	1–11 MPs/100 g	[41]
Yoghurt	NR	Turkey	Multi-enzyme and tetramethylammonium hydrate digestion (40 °C for 24 h) and filtration	1 µm	SEM and ATR-FTIR	Fibers and fragments	2–58 MPs/100 mL	[34]
Breastmilk	NR	Italy	Filtration and digestion with 10% KOH (40 °C for 48 h)	1.6 µm	Raman microspectrometer	Fragment (2–12 µm)	3–11 MPs/L	[33]
Liquid and powder milk	NR	Switzerland	Enzyme (Prozyme) and 25% tetramethyl ammonium hydroxide digestion (80 °C for 1 min)	5 μm	µRaman and optical microscopy; SEM-EDX	Fragments	204–1004 MPs/100 mL	[10]

MPs—Microplastics, NR—Not reported, FTIR—Fourier transform infrared, SEM—Scanning electron microscopy, EXD—Electron-based dissociation, KOH—Potassium hydroxide, H_2_O_2_—Hydrogen peroxide.

## 7. Potential Transformation of NMPs during Food Digestion

The GIT is a targeted organ by NMPs. Factors including the size, shape, distribution, weathering, surface coating, microbial contaminants, adsorbed chemicals, pH, transit time, and redox potential of the GIT may affect the properties of NMPs [42]. Given the series of processes ingested food undergoes in the GIT, knowledge on the potential physicochemical alteration of NMPs during digestion is essential.

In the study of Stock et al. [14], 4 µm MPs (PP, PE, PVC, PS, and PET) of 10, 50, and 100 mg/mL concentrations resisted decomposition by the artificial digestive juices and changes to their shape, size, or texture. The authors suggested that the human GIT primary stages may not transform the MPs; however, biological components such as proteins, lipids, and mucins may adsorb on the MPs, affecting the interpretation of the particle size and shape results. For instance, Tan et al. [12] observed the agglomeration of lipids around MPs and established that MPs affected the digestion of lipids. This consideration is crucial for correctly interpreting the effect of the GIT environment on the MPs.

Through the use of an in vitro Caco-2 monolayer digestive model, Liu et al. [43] attempted to follow the transport of PS-based NMPs (5 nm and 5 µm) and observed that, although the NMPs showed deteriorative effects to the intestines’ physical barrier, the chemical composition of the NMPs was not changed by the digestive process. A study on the physicochemical properties of MPs from the GIT of fish revealed minor changes in weight, size, and colour [44]. When they investigated the potential alteration to the physicochemical properties of polystyrene NPs (50, 300, and 400 nm) and MPs (4 µm), Meng and colleagues found out that the NMPs could not be depolymerized in the mouth, stomach, and intestine and retained their original shape and components [16]. The authors attributed these findings to the high inertia and stability of the NMPs. In the same study, the surface chemistry and the electrostatic adsorption of the NMPs changed, and agglomeration (NMPs–organics, NMPs–NMPs, and NMPs–organics–NMPs) was observed after the GIT digestion. Moreover, the particle size and charge of the NMPs increased and decreased, respectively, a phenomenon the authors attributed to the interaction of NMPs with biomolecules (proteins, lipids, and nucleic acids) in the GIT [16].

Tamargo et al. [45] compared PET–MPs (0.166 g/intake) before and after in vitro GIT digestion and in vitro colonic fermentation and observed that the MPs’ particles maintained their morphology during gastrointestinal digestion and colonic fermentations; however, crystalline and organic matter deposits were observed after small intestine digestion. Furthermore, dynamic in vitro colonic fermentations significantly transformed the MPs’ surfaces, and organic matter was deposited around the MPs. The authors postulated that MPs may alter the composition of the human microbial colonic community and form biofilms in the GIT. According to obtained Raman spectra, there is a trend of structural degradation of the PET MPs during gastrointestinal digestion. FTIR analysis of PP–MPs (spherical, 11.86–44.62 μm) from *P. paludosa* snails showed that PP–MPs did not change during ingestion or after egestion [46].

## 8. Interaction and Effect of NMPs on the Digestion and Absorption of Food Macro-Components

The human digestion system is not capable of decomposing NMPs due to their inertness; thus, the interaction of NMPs with food nutrients is highly possible, and this might affect the digestion and absorption of nutrients by the human body. Despite the huge amount of evidence on the existence of NMPs in food products, information on their potential influence on the digestion and absorption of nutrients is still limited. But available in vitro and animal studies have revealed that NMPs potentially influence the digestion and absorption of carbohydrates, fats, and proteins (Table 3). In addition, the presence of MPs in the GIT could interact with the digestive enzymes [12].

### 8.1. Carbohydrates

The effect of MPs on starch has been widely studied using mussels. O’Brien et al. [11] studied the effect of polystyrene MPs (10 μm) on the digestion of starch using blue mussels (*Mytilus galloprovincialis*). The authors established that MPs (55,000–110,000/L) affected the ability of mussels to digest starch by negatively affecting the amylase enzyme activity (Table 3). It was suggested that MPs negatively influenced the starch digestion in mussels, although the mechanism is not clearly understood. Consistent controls between experiments for factors such as the species, MPs type, exposure duration, food type and level, and temperature are necessary.

Wang et al. [47] reported a decrease in digestive gland amylase activity in hard-shelled mussels exposed to polystyrene MPs (2 μm, >10,000/L) spheres. The elevated gene expression of key energy metabolism genes, which included pyruvate kinase and succinate dehydrogenase, was observed in the digestive glands of mussels contaminated with polystyrene MPs [48]. The authors suggested that this could be due to MPs-induced carbohydrate oxidation. However, studies on other types of carbohydrate enzymes using human digestion models are warranted in order to understand the consequences of MPs regarding nutrient digestion in humans. Although the interaction of starch with MPs has been suggested, the mechanism is still not well understood. 

### 8.2. Fats

**Figure 2:**
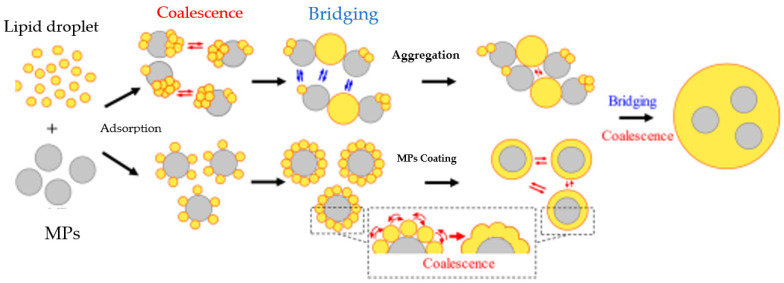
Proposed mechanism for the interaction of lipid droplets with MPs. Reprinted (adapted) with permission from Tan et al. [12]. Copyright (2023) American Chemistry Society. MPs—microplastics.

Five different types of MPs (80 mg/L in the small intestines), including PS, PET, PE, PVC, and PLGA, were reported to significantly reduce lipid digestion in the in vitro gastrointestinal system (Table 3). MPs from PS showed the greatest effect, with lipid digestion decreasing with an increased PS concentration, while the MPs’ size showed no effect. This study demonstrated the need to separately assess the effect of NMPs on nutrient digestion and absorption for each polymer. The study also reported significant interactions of MPs with lipid droplets and lipids [12]. In addition, PS, PE, and PET MPs showed a stronger interaction and aggregation with lipid droplets. Using both experimental and molecular dynamics simulation approaches, two different mechanisms were suggested for the polystyrene MPs-induced lipid digestion inhibition. The first mechanism postulated that the polystyrene MPs decreased the bioavailability of lipid droplets by forming large lipid–MPs heteroaggregrates due to the hydrophobic nature of the MPs (Figure 2). The second mechanism suggested that the MPs adsorbed lipase and reduced its activity by altering its secondary structure [12]. Their findings revealed the potential risk of MPs to nutrient digestion, absorption, and human health.

DeLoid et al. [49] used a three-step in vitro simulated digestion that was combined with a triple culture model to investigate the effect of PE-based MPs on the digestion and absorption of fat. Their study revealed that 400 μg/mL MPs significantly enhanced the digestion and absorption of fat (Table 3). Triacylglycerol (TAG) was depleted, while diacylglycerol (DAG) and intestinal-phase fatty acids were enriched as a result of the pancreatic TAG lipase activity. TAG digestion on the lipid-coated NMPs surfaces was suggested as a potential mechanism. Nevertheless, the proposed mechanism requires further investigation using in vivo studies to establish the NMPs’ properties responsible for their effect on the digestion and absorption of lipids. The enrichment of sterols by over 3% was implicated regarding the addition of bile salts to the small intestinal digestion [49].

A study on lugworms (*Arenicola marina*) showed that lipid and energy reserves were reduced after the lugworms were exposed to 5% PVC-based MPs [50]. The decrease in energy reserves was attributed to the decreased feeding activity, longer ingested material gut residence times, and inflammation. These results are supported by the findings of Sussarellu et al. [51] on oysters, which revealed that MPs (0.023 mg/L) from PS disrupted fatty acid metabolism and reduced energy assimilation. Pollutants attached to the surfaces of the MPs may also affect fatty acid metabolism due to their effect on enzymes such as catalase, allantoinase, uricase, and fatty acid synthase [52].

Overall, it has been highlighted that the disturbance of lipid digestion may cause decreased lipid absorption, insufficient energy intake, decreased essential fatty acids, and fat-soluble vitamins. 

### 8.3. Proteins

Hanachi et al. [52] studied the effects of polystyrene MPs (30 or 300 μg/L) alone and combined with chlorpyrifos insecticide (2 or 6 μg/L) on the digestion of proteins in rainbow trout (*Onchorhynchus mykiss*) (Table 3). MPs alone had a minimal and no effect on amino acids and proteins, respectively. Meanwhile, MPs combined with chlorpyrifos caused a significant decrease in the amino acid and protein contents. The authors suggested that the proteins might have been utilized to provide energy needed to cope with the induced environmental stress. The study highlighted the potential effect of MPs as carriers of toxic compounds and chemicals. Amino acid metabolism disorders were reported in zebrafish (*D. rerio*) exposed to PS–MPs. Leucine, proline, threonine lysine, glutamine, phenylalanine alanine, and tyrosine were considerably altered in the fish’s gut [53]. However, more studies are still needed to investigate the influence of MPs’ size and the dose of individual MPs and MPs combined with other chemical pollutants. 

DeLoid et al. [49], in their study on the effect of PE-based MPs on the digestion of proteins, observed that a total of 72 proteins were depleted in the small intestinal phase. The most notable of these was β-casein, which normally coats and stabilizes milk fat globules and is displaced by bile salts during fat digestion to provide a greater surface area for lipase binding and activity. The depletion of β-casein and the enrichment of triacylglycerol (TAG) lipase in the small intestinal phase were related to the digestion of lipids on the surface of PE by the same mechanism that occurs on fat droplets.

**Figure 3:**
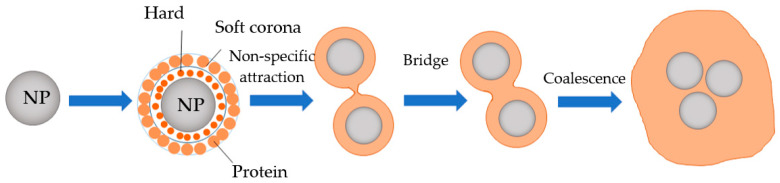
Schematic representation for the interaction of NPs and proteins. Adapted from Gopinath et al. [57].

The adsorption of proteins on MPs, especially on smaller particles, has been reported in the literature [14,54,55,56]. For instance, de Guzman et al. [56] recently observed that cows’ milk proteins (caseins) and their larger fragments preferentially bind to the hard corona of PS–MPs during the gastric digestion of milk, leading to the prolonged survival of larger peptides (particularly of α_S2_-casein) in the simulated gastric fluid. This will be particularly relevant to further studies on the impact of NPs on milk proteins’ digestion and bioavailability. A larger surface area of NPs in comparison to MPs may provide more anchoring points for proteins and digestive enzymes to tightly bind in the hard corona, causing structural changes in proteins and possibly impacting digestive enzymes’ functions. The mechanism for the interaction of proteins with NPs has been proposed [57], with the coalescence of NPs and proteins being the final stage of the interaction (Figure 3).

**Table 3:** Potential effect of NMPs on nutrient digestion using in vitro and in vivo studies.

Nutrient Type	Type of Study	Experimental Model	Key Findings	References
Lipid	In vitro	Stock lipid emulsion (olive oil (4% *w/w*)+ phosphate buffer, pH = 7) was mixed with MPs (PE, PVC, and PET (100, 200, 300, 400 mg/L, and 50 nm, 1 μm, 10 μm)). Lipid digestion was carried out using in vitro simulated digestion.	All the MPs significantly reduced lipid digestion, with PS–MPs exhibiting the highest inhibition. Lipid digestion decreased with an increasing PS concentration. PS-based MPs interacted with both lipid droplets and lipase enzymes.	[12]
	In vitro	A standardized food model (3.4% protein (sodium caseinate), 4.6% sugar (sucrose), 5.2% digestible carbohydrate (corn starch), 0.7% dietary fiber (pectin), 3.4% fat (corn oil), and 0.5% sodium chloride) and high-fat food (33.3% fat) models were mixed with PE–I PM_0.1_. In vitro simulated digestion was performed using a three-phase simulator.	PE–I increased fat digestion and absorption. Fatty acids in the small intestinal phase were enriched.	[49]
	In vivo	Rainbow trout fish (25.1 ± 8.1 g, 9.2 ± 2.2 cm) were exposed to PS–MPs (30 or 300 μg/L), chlorpyrifos (2 or 6 μg/L), or their combination.	The PS–MPs had a minimal effect on fatty acid composition. However, significant alterations in the fatty acid composition were observed in combined PS–MPs and chlorpyrifos.	[52]
Protein	In vitro	A standardized food model (3.4% protein (sodium caseinate), 4.6% sugar (sucrose), 5.2% digestible carbohydrate (corn starch), 0.7% dietary fiber (pectin), 3.4% fat (corn oil), and 0.5% sodium chloride) and high-fat food (33.3% fat) models were mixed with PE–I PM_0.1_. In vitro simulated digestion was performed using a three-phase simulator.	Triacylglycerol lipase enrichment and β-casein depletion were observed.	[49]
	In vitro	Cow’s milk was mixed with PS–MPs (10 µm, 14,200 particles) and subjected to simulated gastric digestion.	PS–MPs promoted the accumulation of larger peptides, suggesting their delayed digestion in the gastric phase.	[56]
	In vivo	Rainbow trout fish (25.1 ± 8.1 g, 9.2 ± 2.2 cm) were exposed to PS–MPs (30 or 300 μg/L), chlorpyrifos (2 or 6 μg/L), or their combination.	The PS-based MPs had no effect on the protein and amino acid contents of fish muscle. Nonetheless, significant changes were observed when PS-based MPs and chlorpyrifos were combined.	[52]
	In vivo	Healthy adult zebrafish (Danio rerio, 5-month-old) were exposed to PS–MPs (5 μm beads; 50 μg/L and 500 μg/L) for 21 days.	Phenylalanine, proline, lysine, leucine, threonine, alanine, glutamine, tyrosine, and ornithine were considerably altered.	[53]
Carbohydrate	In vivo	Mussels of 5–7 cm lengths and 21 months of age were fed with PS spheres (10 μm, 55,000, and 110,000/L).	Exposure to higher levels of PS–MPs raised amylase activity and negatively affected the ability of mussels to digest starch.	[11]
	In vivo	Mussels (M. coruscus (1.5 ± 0.90 g; 7.95 ± 0.32 cm)) were treated with different concentrations of PS microspheres that were 2 mm in diameter (0, 10, 104 and 106/L) at pH 7.7 and 8.1 for 14 days before a recovery acclimation of 7 days.	The alpha-amylase enzyme was significantly inhibited.	[47]

MPs—Microplastics, PS—Polystyrene, PE—Polyethylene, PVC—Polyvinyl chloride, and PET—Polyethylene terephthalate.

## 9. Potential Effect of Ingested NMPs on Nutrient Metabolism

Ingestion has been identified as the primary way by which NMPs enter human bodies among the various possible routes. The GIT should be considered when discussing the potential harmful influence of NMPs, because the ingested NMPs may combine with the physiological functions in the GIT. Millions of NMPs are estimated to be consumed by people every year, usually along with food or water. The yearly human consumption of NMPs could be even higher because not all food groups have been examined for NMPs contamination. Additionally, our exposure data on NPs are lacking, while the exposure data on MPs are incomplete and require a more standardized and comprehensive approach. The intestines are the primary organ for nutrient uptake, metabolism, the immune system, and defence. Data regarding the potential effect of ingested NMPs on nutrient metabolism using dairy products are limited. However, their effects in animals such as mice, fish, bees, and chickens and in vitro studies may help elucidate their potential effects in humans through food consumption (Table 4). 

In the study reported by Li et al. [58], juvenile *M. nipponense* exposed to different PS–NPs concentrations (0–40 mg/L) for 28 days revealed that the content of lactic acid increased as the PS–NP concentration was increased, while the content of glycogen, triglycerides, and total cholesterol decreased. Additionally, the study found that there were significant changes in the expression of the genes for 6-phosphate glucokinase, PK, ACC, HK, fatty-acid-binding protein 10 (FABP 10), CPT-1, and Acetyl-CoA-binding protein (ACBP), which are all involved in metabolism. Ahrendt et al. [59] used juvenile G. laevifrons to study the effects of PSMPs on hyperemia, finding that the effects were more severe at larger doses (0.1 g of PSMPs per 0.5 g of food). When adult zebrafish were exposed to PP–MPs (10 and 100 g/L) for 21 days through commercial food, metabolites were significantly altered by up-regulating glycerophospholipid metabolism and down-regulating fatty acyl metabolism linked to nutritional deficiency [60]. Also, Yin et al. [61] observed abnormal symptoms of the bile, liver, and lumen of the intestine as well as reduced growth and gross in fish exposed to 15 μm PS–MPs. Important liver processes like energy metabolism, glucose metabolism, and fat metabolism can all be destroyed by the effects of NMPs [25]. Similar findings were reported in the studies of Wu et al. [62], Brun et al. [63], Zhang et al. [60], and Lai et al. [64] (Table 4). Asmonaite et al. [65] found different results following a 28-day exposure to 10 mg of PSMPs (500–700 particles per fish per day) in rainbow trout (*Oncorhynchus mykiss*). The authors observed that after the rainbow trout fish ingested PS–MPs, there were no distinct changes in metabolism. It has been suggested that the NMPs’ type, size, and concentration have a significant impact on metabolism, which may account for the variation in the reported findings.

In vivo studies using mice have revealed information about the propensity for NMPs to disrupt metabolism. Okamura et al. [66] fed C57BL/6J (wild-type) male mice that were 7 weeks old a high-fat diet (HFD) along with MPs for 4 weeks. The authors found that mice fed a high-fat diet (HFD) with MPs expressed significantly more genes linked to the Na^+^/glucose cotransporter and long-chain fatty acid transporter than mice fed an HFD alone. Male C57BL/6 mice were administered with polystyrene NPs at doses of 1, 10, and 30 mg/kg/day for eight weeks, either alone or in combination with a diet high in fat and injected with streptozocin (STZ), and they were found to have increased blood sugar, glucose intolerance, and insulin resistance [67]. Moreover, the consumption of PSMPs by mice disrupted the metabolism of protein, lipids, and energy [68,69,70]. Table 4 shows additional studies on the impact of NMPs on metabolism using mice. In chickens, exposure to PS and PE–NMPs increased lipopolysaccharide accumulation, promoted hepatic lipid metabolism disorders, adversely impacted intestinal metabolism and gut microbial homeostasis, and decreased iron absorption (Table 4). Genes related to detoxification and energy balance were depressed when Wang et al. (2022) [71] exposed 10-day-old honeybees (*Apis mellifera*) to PS–NMPs (104 and 105 particles/mL; 100 nm, 1 μm, and 10 μm) through a diet of pollen and 50% sucrose syrup. In vitro studies also confirm the impact of NMPs on metabolism. Increases in amino acids and intermediary metabolites of the tricarboxylic acid cycle were observed in bronchial epithelial BEAS-2B cells treated with 1 mg/mL PS–NPs [72], while lipid accumulation was reported in RAW 264.7 macrophages and BV2 microglial cells after exposure to PS–NPs [73]. Studies by Xia et al. [74] and Palaniappan et al. [75] reported results that were similar (Table 4).

**Table 4:** Potential effect of ingested NMPs on metabolism using in vivo and in vitro studies.

Type of Study	Experimental Model	Key Findings	References
In vivo studies using mice	Five-week-old mice (*n* = 40) were exposed to 0.5 and 50 μm PS MPs (100 and 1000 μg/L) for 5 weeks.	MPs induced gut microbiota dysbiosis and hepatic lipid metabolism disorder.	[76]
	Five-week-old mice were fed with PS–MPs (5 μm, 100 and 1000 μg/L) for 6 weeks.	PS–MPs induced gut microbiota dysbiosis, intestinal barrier dysfunction, and metabolic disorders.	[77]
	Seven-week-old C57BL/6J (wild type) male mice were fed with a high-fat diet together with MPs for 4 weeks.	Mice fed the high-fat diet with MPs showed a higher expression of genes related to the Na^+^/glucose cotransporter and long-chain fatty acid transporter.	[66]
	Forty C57 BL/6 female mice (ICR) and 20 male mice (seven-week-old) were treated with 1 and 10 mg/L polystyrene NPs.	Cholesterol metabolism was disturbed. Metabolic disorders which affected daidzein and sucrose concentrations were reported.	[78]
	Male C57BL/6 mice (six-week-old) were exposed to 100 μg/L or 1000 μg/L MPs, respectively, for 8 weeks.	Exposure to MPs induced the enrichment of genes expressed in the lipid metabolism pathway.	[70]
	PS–NPs were fed to mice at a dosage of 5 mg/kg and 15 mg/kg body weight.	Chronic exposure to PS–NPs increased plasma glucose levels.	[79]
	ICR female mice (7 week old) were administered with MPs (100 and 1000 μg/L) during pregnancy and lactation (∼6 weeks).	MPs caused the metabolic disorder in maternal MPs associated with gut microbiota dysbiosis and gut barrier dysfunction.	[80]
	One hundred male C57BL/6 mice (7–8 weeks old, 20–22 g) were administered for 8 weeks with PS–NPs at dosages of 1, 10, and 30 mg/kg/day alone or combined with a diet high in and injected with streptozocin (STZ).	Insulin resistance, glucose intolerance, and blood glucose level increased.	[67]
	PS–MPs (5 μm and 20 μm fluorescent) were fed to five-week-old male mice (*Mus musculus*, ICR) for 28 days	MPs exposure disturbed energy and lipid metabolism.	[68]
	Sixteen- to twenty-week-old male Hmox1 reporter mice were fed with carboxymethylcellulose (CMC, 0.5% *w*/*v*), a mixture of MPs (1, 4, 10 µm) and CMC (10 mL/kg body weight), for 28 days.	Energy metabolism was impaired.	[69]
In vivo studies using fish	Juvenile M. *nipponense* (22.96 ± 3.87 mm in length and 0.14 ± 0.06 g in weight) were fed for 28 days with NPs of varying concentrations (0, 5, 10, 20, and 40 mg/L).	Metabolism-related genes including 6-phosphate glucokinase, Acetyl-CoA-binding protein (ACBP), HK, CPT-1, PK, ACC, and fatty-acid-binding protein 10 (FABP 10) had their expression altered.	[58]
	Adult marine medaka (*Oryzias melastigma*, 8-month-old) were exposed to 2, 10, and 200 μm PS–MPs at a concentration of 10 mg/L for 60 days.	Exposure to 200 μm PS–MPs increased the bodyweight, hepatic lipid content, and adipocyte size.	[81]
	Juvenile G. *laevifrons* (*n* = 30, body size = 5.0 ± 0.4 cm SL; body weight = 1.5 g ± 0.2 g) were fed with 0.001 and 0.1 g of PS–MPs (8 μm) per 0.5 g of food weekly for 45 days.	Hyperemia was more severe in the higher-exposure group compared to the lower-exposure group.	[59]
	Nile Tilapia (*Oreochromis niloticus*) were fed with 1 mg/L PS NMPs (80 and 800 nm, 8.76 and 80 μm) for 14 days.	An imbalance of gut microbiota homeostasis and disordered liver metabolism were observed in fish fed with 80 nm NMPs.	[62]
	Healthy four-month-old zebrafish (Danio rerio, AB strain, 0.34 ± 0.03 g in wet weight, 33 ± 2 mm in body length) were exposed to pristine MPs (20 mg/L) for 24 h.	Increased metabolism disruption was observed.	[53]
	A total of 100 individuals of M. galloprovincialis (size 4.1 cm ± 0.9 SD) exposed to MPs were subjected to a synthetic polymer powder HDPE (1–50 μm) for 18 days.	Immune-related proteins were produced and growth energy was decreased.	[48]
	Large yellow croaker juveniles (about five months old) were fed with PS NPs suspensions of 0, 1, 10, and 100 mg/kg, respectively, for 21 days.	Liver lipid accumulation was observed. Fatty acid composition changes and lipid metabolism disruption were also observed.	[64]
	Zebrafish wild-type (AB/TL strain) larvae were exposed to 0, 0.2, 2, and 20 mg/ L PS–NPs.	PS–NP-induced disruption of glucose homoeostasis.	[63]
In vivo studies using chickens	Sixty-one-day-old healthy Arbor Acres chickens (48 ± 4 g) were exposed to PE–MPs (200 mg/kg) in feed for 28 days.	PE–MPs exposure negatively affected gut microbial homeostasis and intestinal metabolism.	[82]
	One-day-old (120) chickens were fed with polystyrene-based MPs (1, 10, and 100 mg/L) for 6 weeks	PS–MPs promoted lipopolysaccharide accumulation and promoted hepatic lipid metabolism disorders.	[83]
	Cornish-cross broilers were fed for 14 days with PS (2 mg/kg 50 nm) and carboxylated and undyed NPs.	Lower iron absorption was observed more in chickens exposed to carboxylated NPs.	[84]
In vivo studies using bees	10-day-old honeybees (*Apis mellifera*) were exposed to PS NMPs (104 and 105 particles/mL; 100 nm, 1 μm, and 10 μm) through a diet of pollen and 50% sucrose syrup.	Immune inhibitory genes were stimulated, while genes related to energy balance were depressed.	[71]
In vitro studies	In vitro simulated digestion models for gastric (6 mg PS–MPs were dispersed in 35 mL of gastric fluid; 0.1, 1, and 10 μm) and intestinal digestion were applied.	No significant effect on nutrient absorption or metabolism was observed.	[85]
	RAW 264.7 macrophages and BV2 microglial cells were exposed to 200 nm NPs (1, 5, 10, 25, 50, 100, and 200 μg/mL) before incubation for 24 h.	The exposure of BV2 microglial cells to PS–NPs induced lipid accumulation.	[73]
	A549, HePG-2, and HCT116 cells were treated by 30 nm PS–NPs (25 μg/mL) and 30 nm Au–NPs (0.7875, 1.575, 3.15 ng/mL).	Distribution of cytokinesis-associated proteins was observed.	[74]
	Epithelial kidney cells and L929 mouse fibroblast cell lines were exposed to clear PE (1.0–4.0 μm) and PS (9.5–11.5 μm) microspheres.	The metabolic rate increased as the concentrations of PS and PE–MPs increased.	[75]
	Bronchial epithelial BEAS-2B cells were treated with 1 mg/mL PS–NPs.	Increases in amino acids and tricarboxylic acid cycle intermediate metabolites were observed.	[72]

PS—Polystyrene, NMPs—Nano- and microplastics, MPs—Microplastics, NPs—Nanoplastics, HDPE—High-density polyethylene, PE—Polyethylene.

## 10. Migration of Plastic Oligomers and Their Potential Effect on Health

Apart from NMPs and food additives, NIAS have also been found to migrate from food contact material (FCM) into food or food simulants. In the literature, common representatives of NIAS are plastic oligomers, which are products of incomplete polymerization, degradation, or impurities in the raw material used for production. In contrast to additives, the permitted content of oligomers in food and foodstuffs does not have a defined specific migration limit (SML). Generally, in cases when a substance is not on the positive list according to the European Regulation (EU) No. 10/2011, its maximum migration limit is 10 µg/kg (EC, 2011) [86]. Additionally, other methods of risk assessment can be used. According to the Threshold of Toxicological Concern (TTC), for example, cyclic PET oligomers can be sorted into Crammer Class III substances, which are linked to a higher possibility of a toxic effect, while linear oligomers fall under Crammer Class I, showing little to no toxic effect [87]. Because of the abundance of PET and modified PET in the environment, their oligomers are some of the most often studied.

Food simulants are widely used in migration studies as replacements for food. Linear and cyclic PET oligomers reportedly migrated from teabags into different food simulants, under conditions mimicking tea preparation (5 min, 100 °C). 50% ethanol was used to mimic the addition of milk or cream to the tea. A higher amount of ethanol content was implicated in the higher migration rate of cyclic oligomers for all tested samples, and the first-series cyclic trimer was the most abundant under all studied conditions [87]. Similarly, polybutylene terephthalate (PBT) and PET cyclic oligomers were observed to migrate from coffee capsules, with mostly the PBT dimer and PET trimer being the most abundant [88]. Besides PES-based polymers like PET and PBT, polyamide (PA) polymers can also release cyclic oligomers upon contact with food simulants, as demonstrated by Abe et al. [89] and Kappenstein et al. [90], using various kitchen utensils and tea bags made of PA6 or PA66. The migration of PA oligomers from PA film to 10% ethanol is affected by ionic strength, due to their polar nature, as proven by Tsochatzis et al. [91]. As pointed out by the authors, the salinity of a given food, as compared to the fat content, is rarely considered when investigating the migration of substances from FCM. Polyurethane oligomers have been shown to transverse multiple layers into food simulants when studying multilayer packaging, even exceeding the established migration limits for non-listed substances [92,93].

Regarding detection in food, oligomers and other NIAS have been detected in baby food, among which there are the Ɛ-caprolactam oligomer dimer (0.01–0.02 mg/kg), Bis(2-Hydroxyethyl) terephthalate (BHET) (0.03–0.18 mg/kg), neopentyl glycol–adipic acid (NPG-AA) (0.01–1.06 mg/kg), AA–diethylene glycol (DEG) (1.42–5.86 mg/kg), NPG–sebacic acid (0.05–1.36 mg/kg), NPG–suberic acid (SuA) (0.42 mg/kg), 2NPG-2AA (2.00–4.00 mg/kg), and 2AA-2DEG (0.03–1.10 mg/kg) [94]. Most of these oligomers originate from polyurethane adhesives. Interestingly, 29 of the detected oligomers were cyclic, implying a higher toxicity. The migration of oligomers from polyester-phenol-coating into baby food has also been studied, and cyclic polyester oligomers have been found to migrate into commercial and homemade infant food and their migration was highly impacted by the fat content [95].

Research into the migration of oligomers into food or food simulants is still a work in progress. A few studies on the migration of oligomers into milk are available. PA oligomers, PA6 and PA66, have been shown to migrate from common kitchenware to whole milk (3% fat content), where the most abundant oligomer found was the PA66 monomer. However, migration was below the specified migration limit given by the authors, which is not the case when using a simulant for whole milk (50% ethanol). The authors have pointed out that using food simulants might cause overestimation by a factor of three [96]. The overestimation of migration to milk when using the food simulants is also observed for styrene-acetonitrile trimers under hot fill conditions (70 °C, 2 h), where the content of trimers was below the LOD in milk (7 µg/dm^2^ and 4 µg/dm^2^) while reaching levels between 121 and 428 µg/dm^2^ for 50% ethanol [97]. Other oligomers, such as those originating from PBT, have also been found to migrate into milk (218 µg/L), where experiments in 50% ethanol again led to a fourfold overestimation of migration rates under hot-fill conditions [98]. It is proposed that such discrepancies are caused by a change in material in 50% ethanol, while no such change was observed in milk. According to these results, caution should be applied when extrapolating results for the oligomer migration from food simulants to milk. Migration experiments are summarized in Table 5.

Compared to NMPs, studies of oligomers’ impact on nutrition and metabolism are rare. Generally, the toxicity of cyclic oligomers proposed by the TTC has not been confirmed. Djapovic et al. [99] showed that high concentrations of linear PET oligomers can exhibit toxicity towards nematode C. elegans and a human lung fibroblast cell line, suggesting that caution should be applied in dismissing linear oligomers as non-toxic. In another study, cyclic oligomers that are proven to migrate from multilayer packaging material (AA-DEG and AA-DEG-IPA-DEG, IPA—isophthalic acid) exhibited antagonistic activity on androgen receptors at higher concentrations [93]. Moreover, risk assessment is further complicated by evidence indicating that cyclic oligomers are susceptible to transesterification and hydrolysis in weakly acidic food simulants and simulants containing a low amount of ethanol [92,94,95,100], and at elevated temperatures, they even degrade to a precursor level [101]. These findings extend to simulations of digestion. Eckardt et al. [102] investigated the degradation of three cyclic PBT and PET oligomers under simulated intestinal conditions (37 °C, 4 h, pH 7.4). All three cyclic oligomers (PBT cyclic dimer and trimer and PET trimer) underwent a rapid cleavage in the presence of pancreatin-containing enzymes with esterase activity, resulting in the formation of their linear counterparts. These oligomers were further cleaved to shorter oligomer chains, which occurred faster for PET than for PBT. The previously mentioned oligomers AA-DEG and AA-DEG-IPA-DEG hydrolyzed rapidly during gastric and intestinal digestion, resulting in the formation of linear molecules, decreasing their concentration by 43.7% and 95.8%, respectively. Since most linear oligomers are classified as Cramer class I, this would significantly impact their potential toxicity, increasing the allowed threshold from 90 µg/person/per day to 1800 µg/person/per day, leading to more lenient regulation laws. 

Evidently, research into plastic oligomers, their presence in food, and their health impact is still lacking. Recently, the cyclic PET trimer has been discovered in postmortem human blood samples (6.5–23.3 µg/L) [103], prompting urgency for the further investigation of possible adverse effects on human health through a systematic approach.

**Table 5:** Migration of oligomers into food and food simulants.

Material Investigated	Material Composition	Conditions of the Migration Experiment	Key Findings	References
Tea bags	PET	Tea bags, from which the tea was removed prior to the migration experiment, were immersed for 5 min with constant temperature control in boiling water and 20% and 50% ethanol.	In pure water, only cyclic monomers, dimers, and trimer were found, and an up to first-series heptamer was found in 50% ethanol. In all simulants, the first-series cyclic trimer and second-series cyclic dimer were the most dominant. Linear oligomers have also been found. According to exposure assessment, when tea is prepared in water, the TTC threshold is not exceeded when one tea per day is consumed, but the threshold is exceeded for the second0series cyclic dimer in three out of five bags when five teas are consumed. In the same scenario, the EFSA threshold of 50 µg/kg food for the total oligomer migration is exceeded for four out of five tea bags in cases of cyclic PET oligomers.	[87]
Coffee capsules	PET, PBT, PP	In the first experiment, coffee capsules were placed in an instant coffee machine, and either water or 20% ethanol was passed through the capsules at a temperature of 80–85 °C. In the second experiment, an immersion test was performed by immersing the capsules in water at 92–93 °C or in 20% ethanol at 84–85 °C for 5 min.	Migration was higher in the immersion experiment than in the coffee machine one. The cyclic PBT dimer and pentamer had the highest migration level, while for PET, this was the case for the cyclic dimer and trimer. In addition, migration into 20% ethanol was higher than that into water. Consumption of one coffee per day does not exceed the TTC threshold. However, three out of five capsules do exceed the EFSA limit.	[88]
Various kitchen utensils	PA6, PA66	A total immersion method was used. Sesame grinders and cake servers were treated at 60 °C for 30 min in water, 20% ethanol, and olive oil; ladles were treated at 95 °C for 30 min in water, 20% ethanol, and olive oil; and turners were treated at 121 °C for 30 min in olive oil.	Five PA6 cyclic oligomers and three PA66 cyclic monomer and oligomers were detected. High migration levels of the PA66 monomer, dimer, and trimer into 20% ethanol (260 to 1000 µg/cm^2^) were observed.	[89]
Cooking spoons and tea bags	PA6, PA66	Cooking spoons were treated by boiling 3% acetic acid solution for 2 h in three subsequent migration experiments on three subsequent days. Filled or empty tea bags were inserted in cups with boiling water for 10 min.	From the cooking spoons, the PA 6 cyclic dimer up to hexamer contributed the most to the total PA 6 cyclic oligomer migration, while the cyclic PA 66 monomer and dimer contributed more than 90% of all migrated cyclic PA 66 oligomers. For the tea bags, there is no effect of the food matrix on the migration levels. The highest migration was found for PA 6 tetramer (189.6 µg/L). Based on the TTC approach, the threshold for maximum exposure is exceeded by both the cooking spoons and the tea bags.	[90]
Slotted spoons	PBT	Contact with boiling water (100 °C) for 2 h. Repeated three times for the same spoon.	In the first migrate, seven linear and the cyclic dimer were detected at a level of 0.86 mg per item, which decreased to 0.46 mg per item (second migrate) and 0.34 mg per item (third migrate), with the cyclic dimer making up only 15% of the total oligomers. The authors conclude that the migration of PBT oligomers could be considered safe when discussing aqueous foodstuffs.	[101]
Multilayer baby food packaging materials	PET/Al/PE	Baby food from commercial packaging was analyzed for oligomer content. A migration test on packaging was performed using 3% acetic acid and 20% ethanol for 10 days at 40 °C.	In total, 35 out of 39 NIAS detected were polyester oligomers, and 29 were cyclic, and 6 were linear oligomers. Several of the oligomers quantified exceeded the migration level of 0.01 mg/kg in some of the baby food analyzed. The authors give an example calculation on AA-DEG, where the consumption of one baby food pouch resulted in a daily intake that is 30 times higher than the TTC value (0.015 mg for a 10 kg infant).	[94]
Tin plates with Polyester-phenol-coatings	PE	Migration tests into the food simulants 20% ethanol, 50% ethanol, and water were performed in a sterilizer for 1 h at 121 °C with a 0.5 bar nitrogen excess pressure. The same was repeated with two types of commercial and two types of homemade infant food.	The following contents of cyclic polyester oligomers were detected: water—24.3 µg/dm^3^, 20% ethanol—101.2 µg/dm^3^, 50% ethanol—281.7 µg/dm^3^, commercial infant food (0.2% fat)—16.7 µg/dm^3^, commercial infant food (0.6% fat)—34.7 ug/dm^3^, homemade puree (0.2% fat)—11.6 ug/dm^3^, and homemade puree (5.2% fat)—68.4 ug/dm^3^. Both individual oligomers and polyester-based substances in total can be released in quantities of concern.	[95]
Plates	PBT	A migration experiment was performed in 20% ethanol for 30 days at 40 °C and 60 °C	The migration of cyclic and linear PBT dimers was observed at both temperatures, while the migration of the cyclic PBT trimer was detected only at 60 °C. After 3 days, the migration limit of 50 µg/kg was exceeded. There is a possible swelling effect of the studied material in 20% ethanol, which might cause the overestimation of migration levels.	[104]
Flexible multilayer packaging materials used for cured meat	PET//PA//CPP, PET//Al//PA//CPP, joint by PU layers, CPP on the food contact side	A migration test was performed on bags using the food simulants 10% ethanol, 3% acetic acid, and 95% ethanol. Bags were kept at 60 °C for 10 days.	Caprolactam oligomers from dimer to pentamer were detected in 95% ethanol, with the tetramer being the most abundant in both packaging materials. Several cyclic PU oligomers were also detected in concentrations above the migration limit. Generally, migration was higher in 95% ethanol than in the other two simulants. The cyclic oligomer AA-DEG was shown to hydrolyze into its linear form when in contact with 3% acetic acid.	[92]
Slotted spoons	PBT	Contact with boiling water (100 °C) for 2 h. Repeated three times for the same spoon.	In the first migrate, seven linear and the cyclic dimer were detected at a level of 0.86 mg per item, which decreased to 0.46 mg per item (second migrate) and 0.34 mg per item (third migrate), with the cyclic dimer making up only 15% of the total oligomers. The authors conclude that the migration of PBT oligomers could be considered safe when discussing aqueous foodstuffs.	[101]
Multilayer baby food packaging materials	PET/Al/PE	Baby food from commercial packaging was analyzed for oligomer content. A migration test on packaging was performed using 3% acetic acid and 20% ethanol for 10 days at 40 °C.	In total, 35 out of 39 NIAS detected were polyester oligomers, 29 were cyclic, and 6 were linear oligomers. Several of the oligomers quantified exceeded the migration level of 0.01 mg/kg in some of the baby food analyzed. The authors give an example calculation on AA-DEG, where the consumption of one baby food pouch resulted in a daily intake that is 30 times higher than the TTC value (0.015 mg for a 10 kg infant).	[94]
Tin plates with Polyester-phenol-coatings	PE	Migration tests into the food simulants 20% ethanol, 50% ethanol, and water were performed in a sterilizer for 1 h at 121 °C with a 0.5 bar nitrogen excess pressure. The same was repeated with two types of commercial and two types of homemade infant food.	The following contents of cyclic polyester oligomers were detected: water—24.3 µg/dm^3^, 20% ethanol—101.2 µg/dm^3^, 50% ethanol—281.7 µg/dm^3^, commercial infant food (0.2% fat)—16.7 µg/dm^3^, commercial infant food (0.6% fat)—34.7 ug/dm^3^, homemade puree (0.2% fat)—11.6 ug/dm^3^, and homemade puree (5.2% fat)—68.4 ug/dm^3^. Both individual oligomers and polyester-based substances in total can be released in quantities of concern.	[95]
Plates	PBT	A migration experiment was performed in 20% ethanol for 30 days at 40 °C and 60 °C	The migration of cyclic and linear PBT dimers was observed at both temperatures, while the migration of the cyclic PBT trimer was detected only at 60 °C. After 3 days, the migration limit of 50 µg/kg was exceeded. There is a possible swelling effect of the studied material in 20% ethanol, which might cause an overestimation of migration levels.	[104]
Flexible multilayer packaging materials used for cured meat	PET//PA//CPP, PET//Al//PA//CPP, joint by PU layers, CPP on the food contact side	A migration test was performed on bags using the food simulants 10% ethanol, 3% acetic acid, and 95% ethanol. Bags were kept at 60 °C for 10 days.	Caprolactam oligomers from dimer to pentamer were detected in 95% ethanol, with the tetramer being the most abundant in both packaging materials. Several cyclic PU oligomers were also detected in concentrations above the migration limit. Generally, migration was higher in 95% ethanol than in the other two simulants. The cyclic oligomer AA-DEG was shown to hydrolyze into its linear form when in contact with 3% acetic acid.	[92]
Multilayer packaging materials used for biological fluids and food contact	PET/Al/PA/PP/PE, joint by PU	If the packaging was in contact with the biological fluid, a migration test was performed in water for 3 days at 40 °C. For food contact, a material migration test was performed in 10% or 95% ethanol for 10 days at 60 °C, or in the case of pasteurized materials, for 30 min at 121 °C in 95% ethanol.	The migration values of the cyclic oligomers AA-DEG and AA-DEG-IPA-DEG were tested for 20 packaging materials. In most cases, the migration of cyclic oligomers exceeded the migration limit of 0.01 mg/kg. According to the TTC approach, 6 out of 20 materials complied with FDA guidelines, and 5 out of 20 complied with EFSA guidelines.	[93]
Kitchen utensils	PA6, PA66	Migration experiments were performed in either foodstuffs, such as canned beans, chicken soup, whole milk, and sunflower oil, for 30 min at 100 °C or in the simulants 3% acetic acid or 10%, 50% and 95% ethanol. For the 10% ethanol, the same migration conditions were used as those used for food, and for 50% and 95% ethanol, a temperature of 60 °C was used for 2.5 h.	PA66 monomer was the most abundant oligomer found in both simulants and food, except for sunflower oil, where PA6 monomer was more abundant. The migration of oligomers was overestimated compared to that of real food. For the real foods, the sum of all oligomers was below the migration limit (5 mg/kg). The sums of oligomers in sunflower oil, beans, chicken soup, and whole milk were 4.6 mg/kg, 3.1 mg/kg, 4.4 mg/kg, and 3.6 mg/kg, respectively.	[96]
Shot glasses and reusable coffee cups	SAN and ABS copolymers	For reusable coffee cups, migration experiments were performed in water, acetic acid (3%), ethanol (10, 20, and 50%), coconut fat, sunflower oil, cream (30%), butter fat, Miglyol^®^812, and cow’s milk (3.5% fat) at 70 °C or 100 °C for 2 h. Shot glasses were treated with 50% or 70% ethanol for 30 min at 40 °C.	SAn_2_ and S_2_An trimers are the most abundant oligomers extracted from SAN and ABS. Migration was the highest in 50% ethanol, with a decrease from the first to the third migrate. Migration into oils, fats, and dairy was below the LOD. A product of oligomer degradation, AMNC, was also detected in all extracts.	[97]
Kitchenware	PBT	Migration experiments were performed using food simulants (95%, 50%, and 20% ethanol and 3% acetic acid) and sunflower oil and milk at different temperatures.	In general, cyclic oligomers were dominant in all migrates when performing migration experiments of a hot beverage simulation at 70 °C for 2 h. Migration decreased with every consecutive migration experiment. 50% ethanol overestimated the migration into milk by fourfold. In the case of sunflower oil, migration is affected by temperature. According to the authors, one can consume 410 mL of milk before reaching the threshold of concern. Also, 12–15 g of oil might be consumed before reaching the threshold, which should be equivalent to 100 g of french fries.	[98]

PET—polyethylene terephthalate, PA—polyamide, PP—polypropylene, PE—polyester, PU—polyurethane, DEG—diethylene glycol, AA—adipic acid, IPA—isophthalic acid, TTC—toxicological threshold of concern, FDA—Food and Drug Administration, EFSA—European Food Safety Authority, SAN—styrene-acetonitrile, ABS—acrylonitrile-butadiene-styrene, LOD—limit of detection, AMNC—2-Amino-3-methyl-1-naphthalenecarbonitrile, PBT—polybutylene terephthalate.

## 11. Conclusions and Future Perspectives

The most recent data on NMPs’ and oligomers’ presence in dairy products, their impact on nutrient digestion and absorption, and their health implications were reviewed. Most of the studies provided an insight into MPs’ occurrence and effects on nutrient absorption and digestion. Studies are lacking regarding the occurrence and possible effects of plastic oligomers in food packaging, as their possible migration to milk has been shown in food simulants. Various kinds of dairy products, such as skimmed or whole liquid and powder milk and infant formula milk, have been found to contain NMPs of different sizes, shapes, and concentrations. Given how frequently infants consume dairy products and how both their immune and gastrointestinal systems are underdeveloped in comparison to adults, the presence of NMPs in milk products is a major cause for concern. Given this information, the monitoring of NMPs in foods as a quality control and safety measure by food manufacturers should be seriously considered. In addition, most of our in vitro studies are based on models developed for adults and may not properly reflect NMPs’ impact on infants’ digestion, metabolism, and nutrient uptake. Infants have been found to consume the most NMPs through milk products, which calls for the creation of national laws and rules that control the levels of NMPs in foods. However, the NMPs’ complicated compositions, variable sizes, and shapes, which impact the precise measurement of NMPs’ risks, could hinder these efforts. The reported NMPs concentrations in dairy products varied between studies. A better comprehension of the presence of NMPs in dairy products requires investigations using various packaging materials and at different stages of the value chain. In addition, the standardization of NMPs measurement in dairy products may reduce the chances of the under- or overestimation of NMPs and their risk. The GIT environment may not significantly change the shape and size of NMPs, but interactions with nutrients like lipids and proteins may change their weight, surface chemistry, and electrostatic adsorption. Various results have been reported regarding the potential transformation of NMPs in the GIT.

The agglomeration of NMPs–NMPs and NMPs–organic substances has been observed in the GIT. These findings provide a strong indication that NMPs may interfere with the digestion and absorption of nutrients like proteins, carbohydrates, and lipids, resulting in inadequate calorie consumption and a decreased intake of vital nutrients. To fully understand the mechanisms involved in the interaction of NMPs with nutrients, more focus should be given to the concentration, size, shape, and type of NMPs. The impact of NMPs on the digestion and absorption of other nutrients, such as vitamins and minerals, should also be studied. These investigations are necessary in order to comprehend how NMPs affect nutrient digestion, absorption, and health. NMPs may interfere with the GIT’s normal lipid, protein, glucose, iron, and energy metabolism, which could impact body weight and increase the risk of diseases such as diabetes, cardiovascular disease, hypertension, and some cancers. NMPs may also discharge chemical additives and oligomers into food. The attempts to precisely determine the toxic effects of the substances have been hampered by a lack of chemical standards. Human in vitro models must be used in addition to animal research conducted in vitro and in vivo to better understand how NMPs affect human health. Particular attention should be given to infants, the elderly, and groups already showing digestion impairment, as most of the studies conducted so far consider only healthy adult digestion.

## Data Availability

All data is made available in the manuscript.
